# Stochastics of Degradation: The Autophagic-Lysosomal System of the Cell

**DOI:** 10.32607/actanaturae.10936

**Published:** 2020

**Authors:** A. A. Kudriaeva, A. V. Sokolov, A. A. Jr. Belogurov

**Affiliations:** M.M. Shemyakin and Yu.A. Ovchinnikov Institute of Bioorganic Chemistry, Moscow, 117997 Russia; Lomonosov Moscow State University, Moscow, 119991 Russia

**Keywords:** autophagy, lysosome, autolysosomal degradation

## Abstract

Autophagy is a conservative and evolutionarily ancient process that enables the transfer of various cellular compounds, organelles, and potentially dangerous cellular components to the lysosome for their degradation. This process is crucial for the recycling of energy and substrates, which are required for cellular biosynthesis. Autophagy not only plays a major role in the survival of cells under stress conditions, but is also actively involved in maintaining cellular homeostasis. It has multiple effects on the immune system and cellular remodeling during organism development. The effectiveness of autophagy is ensured by a controlled interaction between two organelles – the autophagosome and the lysosome. Despite significant progress in the description of the molecular mechanisms underlying autophagic-lysosomal system (ALS) functioning, many fundamental questions remain. Namely, the specialized functions of lysosomes and the role of ALS in the pathogenesis of human diseases are still enigmatic. Understanding of the mechanisms that are triggered at all stages of autophagic- lysosomal degradation, from the initiation of autophagy to the terminal stage of substrate destruction in the lysosome, may result in new approaches that could help better uderstand ALS and, therefore, selectively control cellular proteostasis.

## INTRODUCTION

Protein degradation is one of the main functions of the intracellular mechanism, which regulates many important processes, thereby ensuring cellular homeostasis and survival of the whole organism. The autophagic- lysosomal (ALS) and ubiquitin-proteasome (UPS) systems are the main intracellular proteolysis pathways, a decrease or increase in the effectiveness of which significantly affects cellular metabolism in health and disease [1].

**Fig. 1:**
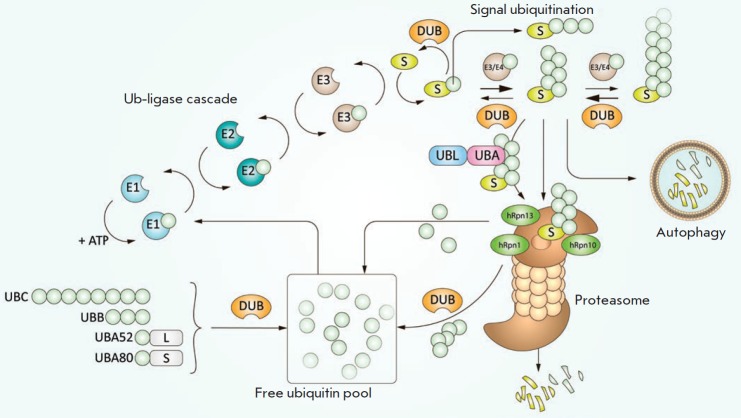
Ubiquitin-proteasome system. Ubiquitin is synthesized in the form of four precursor proteins, UBC, UBB, UBA52, and UBA80, which are further processed by specialized deubiquitinating enzymes (DUBs)–ubiquitin-isopeptidases. The ubiquitination system, which includes three types of ubiquitin ligases (E1 (two enzymes), E2 (tens of enzymes), and E3 (hundreds of enzymes)), is highly specific and selective due to its hierarchical structure. Ubiquitin is conjugated to a substrate (S) as a monomer or a polyubiquitin chain that is formed through the internal lysine residues of the preceding Ub. The polyubiquitin chain is elongated by E3 ligases or the relatively recently discovered ubiquitin ligases of the E4 family. There is a dynamic equilibrium between ubiquitination and removal of ubiquitin residues by ubiquitin isopeptidases, which controls the optimal chain length that amounts, according to modern concepts, to about six ubiquitin molecules per substrate molecule [8]. Further, the ubiquitinated substrate binds to the Rpn10, Rpn13, and Rpn1 proteasome subunits, either directly or with the participation of shuttle proteins of the UBL–UBA family. There may be specific autophagy. A certain amount of ubiquitin enters the proteolytic chamber together with the substrate, which leads to its degradation. In most cases, proteasome deubiquitinase Rpn11 successfully removes the entire polyubiquitin chain that is further cleaved into monomers for recycling

Controlled proteolysis of short-lived and misfolded intracellular proteins occurs mainly in UPS. This system relies on the coordinated actions of three closely related enzymes: the E1, E2, and E3 ligases, which conjugate a small protein ubiquitin (Ub) with polypeptide substrates subjected to degradation [2] (*Fig. 1*). A multi-subunit proteolytic complex called the 26S proteasome recognizes the modified protein. After substrate binding, the ubiquitin chain is released by the deubiquitinating enzyme (DUB) associated with the proteasome; then, the substrate unfolds and is translocated into the inner proteasome cavity, where it is cleaved into short peptides that can be exposed on the cell surface or further degraded to free amino acids by various aminopeptidases [3]. In recent years, evidence of ubiquitin-independent protein hydrolysis has accumulated [4]. Ornithine decarboxylase was the first protein for which a similar degradation mechanism was demonstrated [5]. Recently, a new mechanism of charge-mediated ubiquitin-independent protein hydrolysis by the proteasome was demonstrated for the basic myelin protein, one of the main autoantigens in multiple sclerosis [6, 7].

The main difference between ALS and UPS is that ALS is involved in the degradation of large and potentially dangerous cellular structures, such as protein aggregates and organelles. In most cases, an ALSmediated proteolytic process (also called autophagy) is activated in response to the lack of nutrients in the cell and the proteins of the autophagy-related protein (ATG) family play a significant role in this process [9]. The most studied autophagosomal process is macroautophagy, where cellular components destined for degradation are captured by autophagosomes. Autophagosomes are bilayer membrane vesicles that form from precursors called phagophores–membrane-covered cytoplasm regions that emerge and elongate through the orchestrated action of ATG proteins [10]. Further, autophagosomes merge either directly with lysosomes, where their contents are hydrolyzed by proteolytic enzymes, or first merge with endosomes, forming an intermediate compartment called the amphisome. Inside the lysosome, cytoplasmic material breaks down into metabolites that can be recycled by the cell as building blocks for the synthesis of new macromolecules or as an energy source. Therefore, autophagy is crucial for cell metabolism, especially under conditions of starvation. Also, the removal of damaged or surplus organelles, protein aggregates, and pathogens promotes a longer cell life [11]. Initially, the autophagy process was thought to be non-selective. But later, it became clear that a modification of compounds with ubiquitin, as in UPS, can serve as the degradation signal [12].

UPS or ALS dysfunction can be both the main cause and the result of many pathological processes. Aging, neurodegenerative diseases (Alzheimer’s disease (AD), Parkinson’s disease (PD), and Huntington’s disease (HD)), cardiovascular diseases (including atherosclerosis), cancers, immune system diseases (including rheumatoid arthritis), and muscular dystrophy are directly associated with intracellular proteolysis impairment [13]. In this regard, knowledge of the molecular ALS machinery, and the pathways of its regulation, becomes especially important.

## MECHANISMS AND TYPES OF AUTOPHAGY

Autophagy is an evolutionarily ancient catabolic process, the mechanism of which is conservative in all eukaryotic cells, from yeast [14] to mammals [15]. Basal (unstimulated) autophagy occurs in all cells at a consistently low rate, but can be activated in cases where cells need nutrients and energy (e.g., during starvation), in the remodeling of existing or elimination of harmful cytoplasmic components (e.g., during oxidative stress, infection, or ER stress-induced protein accumulation). Autophagy mediates the degradation of oxidized lipids, damaged organelles (e.g., mitochondria and peroxisomes), and intracellular pathogens (bacteria and viruses). Autophagy is involved in the degradation of aggressive aggregates of cytoplasmic proteins in neurodegenerative diseases, e.g., various dementia forms (caused by the tau protein), Parkinson’s disease (α-synuclein), and Huntington’s disease (mutant huntingtin). Autophagy protects against certain infectious diseases caused, e.g., by *Salmonella typhimurium *and *Mycobacterium tuberculosis*. Degradation of stored material produces nucleotides, amino acids, and free fatty acids, which are used to synthesize macromolecules and ATP. Finally, autophagy protects cells from age-related changes. Therefore, this complex process regulated by many factors is involved in the protection of cells from malignant transformation, infectious diseases, as well as metabolic, muscular, inflammatory, and neurodegenerative disorders.

As mentioned in Introduction, autophagy was initially considered a non-selective degradation process. However, it soon became apparent that autophagy may be very selective. Despite the growing list of substrates selectively degraded by autophagosomes, the exact mechanisms underlying substrate recognition in autophagy remain poorly understood. In the deficiency of nutrients or growth factors, autophagy is a non-selective process. Selective and non-selective autophagy processes are triggered by various signals. However, all initiate the membrane remodeling necessary for the autophagosome formation.

To date, three autophagy types have been identified: microautophagy; chaperone-mediated autophagy (CMA), which is found only in mammals; and macroautophagy.

**Fig. 2:**
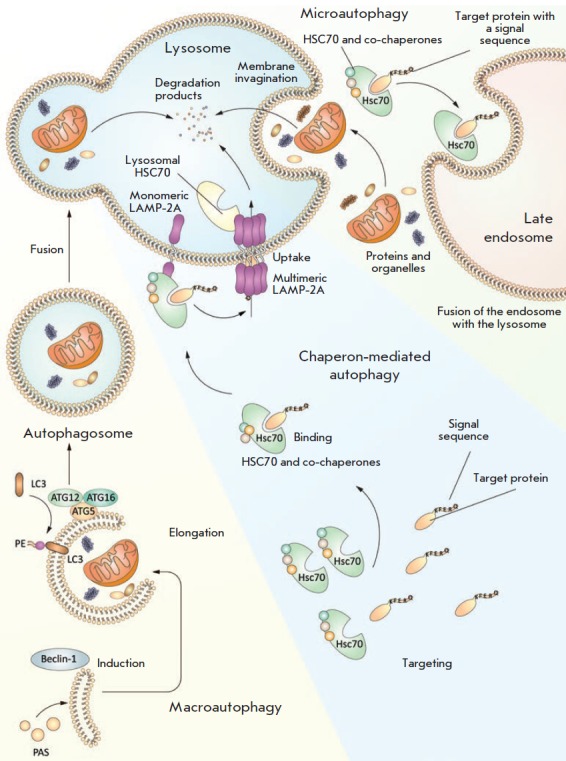
Autophagy types. The least studied autophagy type, microautophagy, promotes direct delivery of organelles and other cellular components to lysosomes. In chaperone-mediated autophagy, cargo recognition by the HSPA8/HSC70 chaperone occurs due to the presence of the KFERQ signal pentapeptide in the cargo sequence. Chaperone with the cargo binds to the lysosomal membrane protein 2A (LAMP2A), and the cargo is translocated into the lysosomal cavity. In macroautophagy (or autophagy), ATG proteins are recruited to the phagophore assembly site (PAS), where the isolation membrane, which forms the phagophore, originates. Elongation of the curved isolation membrane and its further closure leads to the formation of double-membrane vesicles, autophagosomes, that uptake cellular material. Then, the autophagosome merges with the lysosomal membrane to form the autolysosome. This fusion leads to the degradation of the autophagosome, together with cellular material in the lysosomal cavity

Microautophagy is the least studied type of autophagy (*Fig. 2*). This autophagy is subdivided into three types: microautophagy, with lysosomal protrusion (type I); microautophagy, with lysosomal invagination (type II); and microautophagy, with endosomal invagination (type III) [16]. Type I microautophagy involves the ATG5 (in plants) and Vac8, and ATG18 (in *Pichia pastoris *yeast) proteins. Type III microautophagy was identified relatively recently and was studied in a mouse dendritic cell line and *Drosophila melanogaster*. This microautophagy also involves some of the proteins of the endosomal sorting complex required for transport (ESCRT), such as Nbr1 and HSC70. In general, microautophagy facilitates the direct delivery of organelles and other cellular components to lysosomes: e.g., peroxisomes (micropexophagy), nuclear components (piecemeal microautophagy of the nucleus), and mitochondria (micromitophagy). This type of autophagy can be activated not only under conditions of starvation, but also under normal conditions, with intact cell components being degraded.

In chaperone-mediated autophagy, cytosolic proteins containing a specific signal sequence, the KFERQ pentapeptide, are recognized by the 70 kDa heat shock protein (HSPA8/HSC70) that, in turn, binds to the lysosomal membrane protein 2A (LAMP2A). Next, the target proteins undergo unfolding and are translocated to the lysosomal lumen, where they are degraded [17] (*Fig. 2*).

Induction of macroautophagy (hereinafter simply referred to as autophagy) is accompanied by the recruitment of ATG proteins to the phagophore assembly site (PAS), which is a cup-shaped isolation membrane. Gradual elongation of the curved isolation membrane leads to phagophore expansion. Finally, the membrane closes, resulting in double-membrane vesicles–autophagosomes. The sizes of the autophagosome vary within 0.5–1.5 μm, depending on the autophagy-inducing signal, cargo to be degraded, and cell type [11]. After delivery via microtubules to the lysosome, the autophagosome membrane fuses with the lysosomal membrane to form the autolysosome. This fusion leads to the degradation of the autophagosome, along with the cargo present in the lysosomal cavity (*Fig. 2*).

## AUTOPHAGIC STAGES

**Fig. 3:**
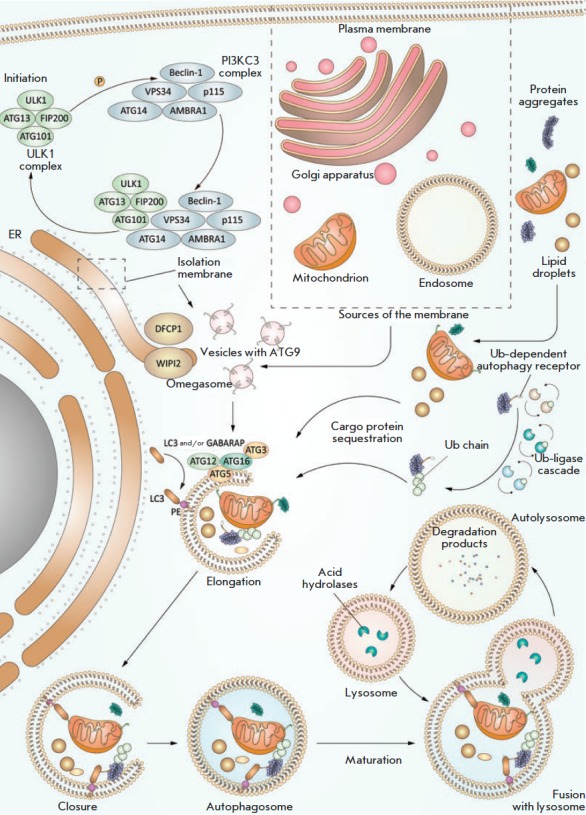
lysosomal system. Autophagy is initiated under various stress conditions, such as starvation, hypoxia, oxidative stress, protein aggregation, endoplasmic reticulum stress, etc. The main initiator complex, ULK1, which consists of the proteins ULK1, ATG13, FIP200, and ATG101, initiates phagophore nucleation using class III phosphatidylinositol-3-kinase complex I (PI3KC3– C1) comprising ATG14, Beclin1, Vps34, and AMBRA1, as well as the vesicular transport factor p115 that activates the production of phosphatidylinositol- 3-phosphate (PI3P) in the omegasome – a subdomain of the endoplasmic reticulum membrane. Then, PI3P recruits the WIPI2 and DFCP1 proteins to the omegasome via their interaction with PI3P. Recently, WIPI2 was shown to directly bind to ATG16L1, recruiting the ATG12–ATG5– ATG16L1 complex that enhances ATG3-mediated conjugation of ATG8 family proteins, including the LC3 and GABARAP proteins, with phosphatidylethanolamine (PE), thus producing membrane- bound lipid forms. ATG8 not only additionally recruits autophagosomal machinery components containing the LC3-interacting region (LIR); it is also necessary for the elongation and closure of the phagophore membrane. In selective autophagy, LC3 is involved in the sequestration of labeled cargo into autophagosomes through LIR-containing cargo receptors. Some cell membranes, including the plasma membrane, mitochondria, endosomes, and the Golgi apparatus, promote the elongation of the autophagosome membrane by transferring their own membrane material (some of these lipid bilayers are delivered by ATG9-containing vesicles, but the origin of the remaining lipid bilayer is currently unknown). Closure of the autophagosome membrane leads to the formation of a bilayer vesicle called the autophagosome that matures (ATG proteins are removed) and finally merges with the lysosome. Lysosomal acid hydrolases degrade autophagic cargo, and then nutrients are released into the cytoplasm for recycling

The autophagy process includes several stages: initiation, autophagosome formation, expansion and elongation of the autophagosome membrane, membrane closure, fusion of the autophagosome and the lysosome, and content degradation (*Fig. 3*) [18].

**Initiation**

The initiation stage is regulated by various proteins, depending on the initial signal inducing autophagy. These include four protein kinases: mTORC1, ULK1, AMPK, and AKT. Starvation is one of the most studied autophagy induction factors; in this case, mTOR serine/threonine kinase, which is part of the mTORC1 complex, plays a significant role in determining the availability of nutrients. A lack of nutrients, mainly amino acids, triggers a signaling cascade that inhibits mTORC1 activity [19]. Inactive mTORC1 dissociates from ULK1, which leads to dephosphorylation and activation of the ULK1 complex (also known as ATG1) comprising the ULK1, ULK2, ATG13, FIP200 (RB1CC1), and ATG101 proteins. The ULK1 complex initiates the phagophore formation by phosphorylation of the components of class III phosphatidylinositol- 3-kinase complex I (PI3KC3–C1) that contains the proteins VPS34/PIK3C3, ATG14L, AMBRA1, and Beclin 1 (ATG6 ortholog) and the transport factor p115 that activates the formation of phosphatidylinositol- 3-phosphate (PI3P) in the omegasome, a compartment of the endoplasmic reticulum.

**Membrane growth**

After ULK1 complex formation, class III phosphatidylinositol- 3-kinase complex I (PI3KC3–C1) is recruited to the phagophore. The PI3K complex is necessary for the nucleation and assembly of the isolation membrane. Its main component, the VPS34 protein (a catalytic subunit of the PI3K complex), is recruited by ULK1 and produces PI3P at the initiation sites. PI3P is critical for the formation of autophagosomes and is considered a marker of autophagosome membranes.

Phosphatidylinositol-3-phosphate, which is produced at the phagophore formation site, provides a platform for recruiting downstream autophagosomal effectors, such as the WIPI and ATG16L family proteins. The WIPI2 protein directly binds to ATG16L1, recruiting the ATG12–ATG5–ATG16L1 complex that promotes conjugation of ATG8 ubiquitin-like (UBL) family proteins, which include the LC3 and GABARAP proteins, with phosphatidylethanolamine (PE) located on the phagophore membrane, forming membranebound lipidated protein forms. The modified ATG8 proteins additionally recruit components containing the LC3-interacting region (LIR), which promotes the elongation and closure of the phagophore membrane. A lipid-conjugated form of the LC3 protein may be considered an autophagosome marker. In addition, in selective autophagy, LC3 is involved in the delivery of specifically labeled cargo to autophagosomes via LIRcontaining cargo receptors.

It is important to note that the Golgi apparatus, plasma membrane, and endosomes can also participate in autophagosomal biogenesis [11], promoting autophagosome membrane elongation by donating membrane material (some lipids are delivered by ATG9-containing vesicles, but the origin of the remaining lipid bilayer is unknown at this time).

**Autophagosomal cargo recognition**

Selective autophagy is of fundamental significance in cell metabolism. In selective autophagy, receptors recognize cargo and attach it to a nascent autophagosome (*Fig. 3*). The receptors comprise the LC3-interacting region that contains the Trp/Phe/Tyr-xx-Leu/Ile/ Val (W/F/YxxL/I/V) consensus sequence binding to the UBL proteins LC3/GABARAP exposed on the autophagosome membrane [20, 21]. Recent data indicate that cargo-bound autophagosomal receptors can locally initiate autophagy by recruiting and activating the most important components of the autophagosomal system (e.g., the ULK1 complex) [22, 23]. This differs from the autophagy caused by nutrient deficiency, where initiation of autophagy and formation of the autophagosome membrane are not dependent on the cargo but are regulated by protein kinases [11].

Depending on the type of uptaken cellular material, selective autophagy is subdivided into aggrephagy (aggregated proteins), mitophagy (mitochondria), pexophagy (peroxisomes), lipophagy (lipid droplets), ribophagy (ribosomes), reticulophagy (ER), xenophagy (pathogens), glyophagy (glycogen), zymophagy (zymogen), nucleophagy (nucleus), chromatophagy (chromatin), myelinophagy (myelin), ferritinophagy (ferritin), lysophagy (lysosomes), granulophagy (stress granules), and proteaphagy (proteasome) [16, 17].

In addition to binding to autophagosome membranes, the receptors should recognize cargo, i.e. distinguish normal organelles or cellular structures from damaged or surplus ones [24]. In higher eukaryotes, binding may have to do with ubiquitination of the cargo. This mechanism is the prevalent form of mammalian cargo recognition [25]. In addition to the Ub-dependent pathway of delivery to the autophagosome, there is also an Ub-independent one. Often, delivery of the same cargo occurs via both mechanisms [20, 25, 26].

**Ubiquitin-dependent autophagy**

Ubiquitinated proteins are known to accumulate during UPS inhibition and form aggregates that are utilized by autophagy [20]. To date, about 20 selective autophagy variants have been reported [19, 23], and almost half of them are Ub-dependent. In Ub-dependent autophagy, the cellular components that are to be delivered to the autophagosome undergo a modification by Ub that, in turn, is recognized by a receptor containing the ubiquitin-binding domain (UBD) [21, 27]. The cell has a large number of autophagosomal receptors for the recognition of intracellular ubiquitinated aggregates (p62, NBR1, OPTN, TOLLIP) [28-32], bacteria (p62, OPTN, NDP52) [33, 34, 35], peroxisomes (NBR1) [36], mitochondria (OPTN, NDP52, Tax1BP1) [23, 37, 38], zymogens (p62) [39], proteasomes (RPN10) [40], equatorial plates (midbody) (p62, NBR1) [41], or nucleic acids (p62, NDP52) [42, 43] and for the binding of cargo to the autophagosome membranes (*Fig. 3*). The ability of ubiquitinated proteins to form aggregates, thereby turning into autophagosomal substrates, is supposed to depend on the Ub chain length and type [44]. There is experimental evidence of increased affinity of K63 polyubiquitinated chains for the p62 and NBR1 autophagosomal receptors [45, 46], while proteins modified with the K48, K27, and K11 chains undergo proteasomal hydrolysis [47].

**Aggrephagy**

**Fig. 4:**
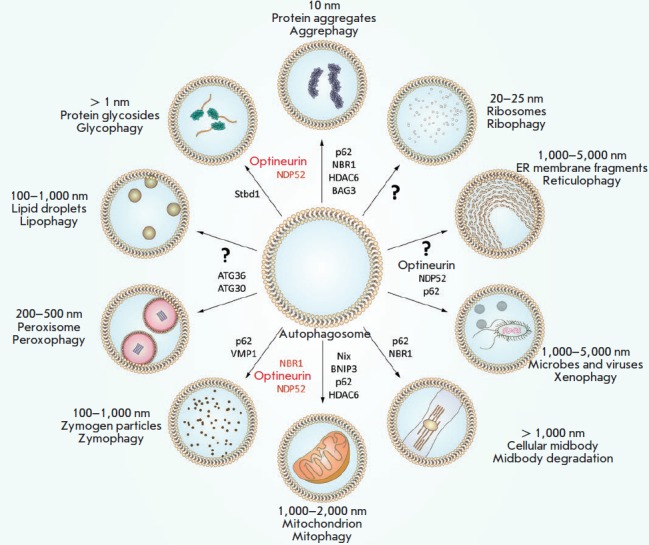
Aggrephagy mechanism–selective degradation of protein substrates through autophagy

Aggrephagy, or selective degradation of protein aggregates by autophagy, is an example of ALS and UPS cross-action (*Fig. 4*). For example, deubiquitinating enzymes (DUBs) are involved in both systems. Autophagy also involves UBL proteins that are recognized by autophagosomal receptors, such as SUMO-1 and FAT10 [48, 49], as well as the UBL protein ISG15 that binds to the HDAC6 and p62 receptors, facilitating lysosomal degradation of protein aggregates [50]. BAG family molecular chaperones, the BAG1 and BAG3 proteins, compete for the polyubiquitinated substrates associated with the chaperones. The BAG1 protein delivers substrates to the proteasome, while BAG3 interacts directly with p62 and simultaneously binds K48 polyubiquitinated chains, directing the proteins initially targeted at the proteasome to degradation into lysosomes [51]. Aggregation-prone proteins, such as β-amyloid [52], huntingtin [53], and α-synuclein [54], are autophagosomal substrates, but according to other data, they can also be degraded by the proteasome. A yeast protein, Cue5, is a receptor that promotes the elimination of aggregates containing proteins with polyglutamine (polyQ) segments. Cue5 contains the CUE ubiquitin-binding domain and the AIM domain mediating the interaction between ubiquitinated cargo and ATG8 proteins [32]. Overexpression of the human TOLLIP protein, a Cue5 homologue, which also has a CUE domain, leads to the degradation of polyQ protein aggregates in human cell lines [55]. In mammals, at least three receptors, SQSTM1 [28, 56], NBR1 [29], and OPTN [57], function as ubiquitin-binding proteins that mediate the interaction between ubiquitinated proteins and the autophagosome machinery. All three receptors have LIR- and ubiquitin-binding domains; i.e., they serve as an adapter between proteins of the LC3/GABARAP family and ubiquitinated substrates. It is assunmed that protein aggregates that cannot be degraded by UPS (e.g., due to size) can be eliminated by autophagy [58].

**Autolysosomal hydrolysis**

**Fig. 5:**
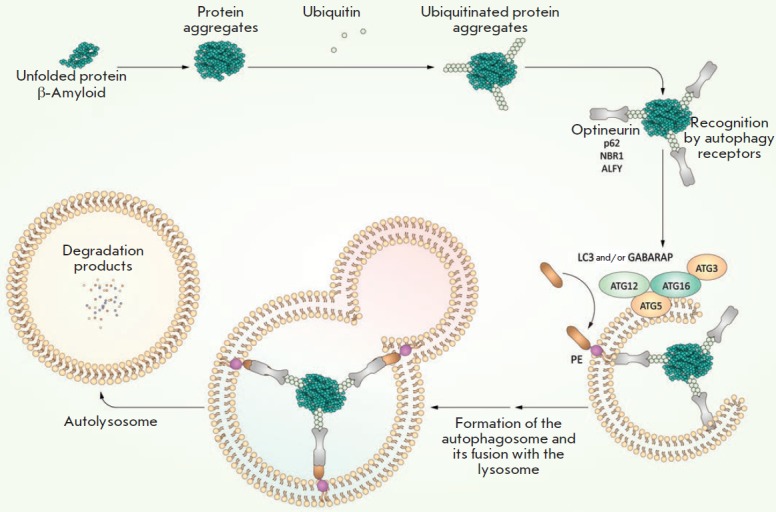
Autophagosomal receptors. Selective autophagy processes are called depending on the type of uptaken cellular material. At present, the receptors of some autophagosomal cargo are unidentified. Individual autophagosomal receptors are involved in the delivery of several cargo types, such as p62 and NBR1

Closure of the autophagosome membrane leads to the formation of a bilayer vesicle called the autophagosome, whose maturation is accompanied by removal of ATG proteins (*Fig. 5*). After this, the autophagosome merges with the lysosome, but the exact mechanism accompanying this process is not clearly understood. RAS-like GTPases and soluble N-ethylmaleimide-sensitive protein receptors (SNARE) are known to be involved in this process [15]. In addition, there is evidence that the microtubule system is necessary for the transfer of mature autophagosomes from random initiation sites to the perinuclear region [59], where they merge with endosomes or lysosomes. In addition, regulation of the transport of mature autophagosomes to lysosomes involves the PI3K complex in which ATG14L is replaced by the UV radiation resistance-associated gene (UVRAG) [15].

After fusion with the autophagosome, lysosomal acidic hydrolases cleave autophagosomal cargo, and then nutrients are released back into the cytoplasm for recycling by the cell. Degradation of cellular material in the lysosome is the final stage of autophagy.

## LYSOSOME

Lysosomes were first described by the Belgian biochemist Christian de Duve in 1955 [60]. They are present in all eukaryotic cells and vary in shape and diameter (from 0.2 to 2.0 μm). At present, the lysosome functions are believed to be broader than previously thought; lysosomes are involved in many fundamental processes, such as regulation of signal transmission, energy metabolism, plasma membrane recovery, regulation of transcription, cell homeostasis, cholesterol transport, and the immune response. Lysosomal functions may be divided into three main types: secretion, signal transmission, and degradation.

**Fig. 6:**
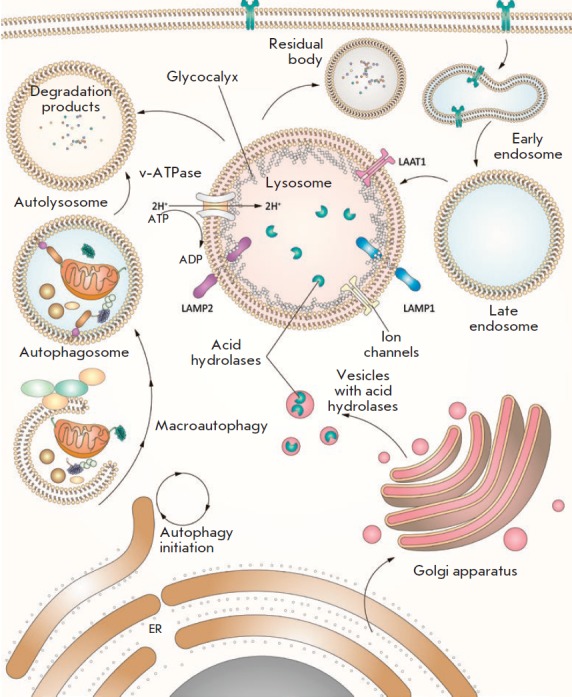
Lysosome in the autophagosomal process. Lysosomes are surrounded by a single-layer membrane containing integral and peripheral proteins. The lysosome comprises an acidic lumen that contains about 60 soluble hydrolytic enzymes and activators. Structural LAMP1 and LAMP2 glycoproteins are the most common lysosomal membrane proteins. Vacuolar-type ATP-dependent proton pumps (v-ATPases) actively function in the lysosomal membrane to maintain the stable acidic environment necessary for the internal hydrolytic activity of the lysosome. Similar molecular pumps are also involved in LYNUS and use the ATP hydrolysis energy to pump protons into the lysosomal lumen. The proton gradient also ensures the transfer of proton-bound metabolites, ions, and soluble substrates in both directions [76] and is necessary for the correct transport of newly synthesized lysosomal enzymes from the Golgi complex to the lysosome

Lysosomes play a central role in the degradation of cellular organelles as well as extracellular and intracellular macromolecules. These organelles have a highly acidic lumen (pH ~4.5–5.0) surrounded by a lipid bilayer, which contains a pool of soluble hydrolases capable of degrading proteins, proteoglycans, nucleic acids, and lipids (*Fig. 6*). The marker enzyme of lysosomes is acid phosphatase. The optimum activity of lysosomal enzymes occurs at pH 5.0; therefore, in a neutral environment, e.g., in the cytoplasm, their activity is greatly reduced, which protects the cell upon accidental release of these enzymes from the lysosome. However, some enzymes, especially those of the cathepsin class, largely retain extra-lysosomal activity: so their release can affect cellular metabolism [61, 62]. The lysosome membrane contains proteins that are involved in the transport of molecules both from the lumen and into it to maintain an acidic environment and also participate in the fusion of the lysosome with other cellular structures. Substrates subjected to degradation enter the lysosome in various ways. Extracellular material subjected to proteolysis is delivered to the lysosome via endocytosis [63], while intracellular components are degraded in lysosomes by autophagy [15]. In addition, lysosomes may be involved in necrosis and apoptosis. Permeabilization of lysosomes and subsequent release of enzymes into the cytosol are considered to be aspects of “lysosomal apoptotic pathway.” Cell death caused by the activity of lysosomal enzymes occurs through apoptosis or necrosis, depending on the permeabilization of lysosomes, namely the number of proteolytic enzymes present in the cytosol [64]. For example, complete organelle degradation with the release of large amounts of lysosomal enzymes causes uncontrolled necrosis, while selective lysosomal permeabilization leads to the induction of apoptosis [65, 66]. Once lysosomal hydrolases are released into the cytosol, they can participate in the apoptotic cascade, acting either in conjunction with the canonical caspase pathway or directly participating in the active cleavage of key cellular substrates [67, 68].

Lysosomes can secrete their contents via lysosomal exocytosis, a process that can be detected based on the translocation of lysosomal membrane markers, such as the lysosome-associated membrane protein (LAMP1), into the plasma membrane [69-71]. This process is most active in some cell types; e.g., in hematopoietic strain cells, osteoclasts, and melanocytes. Lysosomes fuse with the plasma membrane via a mechanism involving the activation of a lysosomal Ca^2+^-dependent channel, MCOLN1, which leads to the release of lysosome contents into the extracellular space [71-73]. This process plays an important role in the recovery of secretion and the plasma membrane. Initially, lysosomal exocytosis was believed to occur only in professional secretory cells containing lysosome-related organelles (LROs) [74], but it was soon shown that any cell type can perform such a function [71]. Lysosomal exocytosis mediates several physiological processes, such as degranulation of cytotoxic T lymphocytes [75], bone resorption by osteoclasts [76], mast cell and eosinophil protection against parasites [77, 78], and the function of melanocytes in pigmentation [79] and platelets in coagulation [80].

The molecular mechanism mediating Ca^2+^-regulated exocytosis of lysosomes involves the VAMP7 protein from the SNARE family, transmembrane Ca^2+^-binding protein synaptotagmin VII (SYTVII), SNAP23, syntaxin 4 [81], as well as several RAB proteins on the lysosome surface [70, 82, 83 ]. Autophagy proteins can also regulate lysosomal exocytosis. For example, lipidation of the autophagosomal marker LC3 is necessary for the secretion of the lysosome contents into the extracellular space, because this targets the lysosome to fuse with the plasma membrane [84, 85]. However, autophagosomes cannot mediate this process [85]. Interestingly, lysosomal exocytosis is controlled by the transcription factor EB (TFEB) that is the main regulator of lysosome biogenesis. TFEB promotes both docking and fusion of lysosomes with the plasma membrane by regulating the expression of certain genes whose protein products contribute to the MCOLN1-mediated increase in the amount of intracellular Ca^2+^ ions [86].

Lysosomal exocytosis is not only responsible for the secretion of lysosomal contents, but also plays a decisive role in plasma membrane recovery. Plasma membrane damage leads to a rapid migration of lysosomes to damaged sites. Then, lysosomes merge with the plasma membrane and effectively seal the damaged sites [87, 88]. This process is especially important in defense mechanisms against bacterial infections [89].

Recently, it has become apparent that the lysosome plays an important role in the determination of the nutrients and in the signaling pathways that are involved in metabolism and cell growth. It is noteworthy that the multi-molecular signaling complex mTORC1, the main monitor of cell and organism growth [90], is activated on the lysosomal surface by growth factors or in response to the accumulation of amino acids [91]. mTOR, the main catalytic component of mTORC1, is an atypical serine/threonine kinase; its functions are often impaired in various diseases, in particular in malignant lesions [92]. Free amino acids were shown to initiate translocation of the mTORC1 complex to lysosomes, where it is activated through interaction with Rag GTPase, as well as the Ragulator and Rheb proteins attached to the lysosomal membrane [91]. Activated mTORC1 is responsible for phosphorylation and subsequent accumulation of the nuclear factor TFEB in the cytosol, thereby transmitting the signals from lysosomes to the nucleus [93].

**Lysosome structure**

Lysosomes are surrounded by a single-layer membrane containing integral and peripheral proteins. Inside the lysosome, there is an acidic lumen containing about 60 soluble hydrolytic enzymes and activators [94], such as sulfatases, glycosidases, peptidases, phosphatases, lipases, and nucleases, which allow the lysosome to degrade an extensive repertoire of biological substrates, including glycosaminoglycans, sphingolipids, glycogen, and proteins [95]. The inner perimeter of the lysosomal membrane is covered by a thick glycocalyx layer that protects the membrane from acidic lumen hydrolases. Soluble lumen enzymes are directly involved in degradation; the lysosome membrane actively participates in the maintaining of the cell membrane integrity, acidity of the lysosomal lumen (pH), and transfer of metabolites, ions, and soluble substrates to and from the lysosome. The structural glycoproteins LAMP1 and LAMP2 are the most common proteins of the lysosomal membrane; they account for more than 50% of the total protein in this membrane, and their expression varies in different tissues, which is an indication of the differences in their functions. These proteins, especially LAMP2, are important regulators of the maturation of phagosomes and autophagosomes, and lack of these proteins disrupts the dynein-driven transport of lysosomes to the perinuclear space, where they merge with autophagosomes [96, 97].

In addition, the LAMP2A isoform is involved in chaperone-mediated autophagy (CMA), a process in which specific proteins are targeted to lysosome degradation via recognition of a specific motif in their amino acid sequence [98].

**Protein composition of lysosomes**

A large number of protein complexes located on the lysosome surface are involved in the mechanism of lysosomal nutrient sensing (LYNUS) (*Fig. 6*). Their role is to directly determine the contents of nutrients (in particular, amino acids) in the lysosomal lumen, as well as to transduce information to the cytoplasm and nucleus. The stable acidic environment necessary for the internal hydrolytic activity of the lysosome is maintained by the vacuolar-type ATP-dependent proton pumps (v-ATPases) located in the lysosomal membrane. Similar molecular pumps are also present in LYNUS and use the ATP hydrolysis energy to pump protons into the lysosomal lumen. The proton gradient also ensures the transport of metabolites, ions, and soluble substrates in both directions [99]; it is necessary for a correct transport of newly synthesized lysosomal enzymes from the Golgi complex to the lysosome. Maintenance of the acidic lysosomal lumen environment may also involve chloride channel (CLC) family proteins, namely CLC7 [100, 101], cation channel mucolipin 1 (MCOLN1, known as TRPML1), and two-pore channels (TPCs) [100] that mediate the transfer of Ca^2+^ and Na^+^ ions from the lysosome. A recently identified lysosomal membrane protein, LAAT1, is involved in the transport of lysine and arginine amino acids from and to the lysosome. This protein apparently plays a decisive role in amino acid homeostasis in the cell [102, 103]. The endolysosomal ATP-sensitive Na^+^ channel (lysoNaATP) located on the lysosomal membrane is also involved in nutrient sensing, regulation of the pH stability of the lysosomal lumen, and amino acid homeostasis, responding to the ATP level and controlling the lysosomal membrane potential [104]. It should be noted that the role of each of these channels and the exact mechanisms underlying the regulation of lysosomal lumen acidification are still poorly understood. Dissipation of the transmembrane proton gradient is known to decrease the efficiency of the transport through the lysosomal membrane, which, in turn, leads to impaired degradation of cellular waste and, ultimately, to metabolic disorders [96].

There have been several attempts to analyze the protein composition of lysosomes [94, 105]. However, the methods for isolating lysosomes from the cell are based either on subcellular fractionation or the specific features of soluble lysosomal proteins, e.g., modification of mannose-6-phosphate (Man-6-P); for this reason, when analyzing the data, it is difficult to distinguish resident lysosome proteins from the proteins directed to the lysosome for degradation. To date, about 100 lysosomal proteins are known; of these, 70 are lysosomal lumen proteins, and about 50 are lysosomal membrane proteins [94]. Obviously, not all lysosomal proteins are identified.

**Lysosome formation**

Primary lysosomes form in the Golgi apparatus region, and lysosomal proteins, in turn, are synthesized and glycosylated in the rough endoplasmic reticulum (RER). Maturation of lysosomal proteins is a specific stage. In a two-step reaction, terminal mannose (Man) residues are phosphorylated at position C6, which occurs in the *cis*-Golgi region. First, N-acetylglucosamine- 1-phosphate is transferred to the OH group at the C6 atom of terminal mannose; then, N-acetylglucosamine is cleaved, and the terminal mannose-6-phos phate group is attached to a protein [106]. It is this modification that underlies the directed transport of lysosomal enzymes to lysosomes as well as their ability secrete. Membranes of the *trans*-Golgi network (TGN) to contain the mannose 6-phosphate receptor (MPR). There are two types of receptor molecules that recognize Man-6-P: cation-independent MPR (CI-MPR) and cation-dependent MPR (CD-MPR). They recognize the lysosomal proteins bearing these groups and bind them. Local clustering of these receptors occurs with the participation of clathrin; therefore, only specific sites of the membrane are removed and transferred by transport vesicles to endolysosomes, whose maturation results in primary lysosomes. Finally, a phosphate group is cleaved from the Man-6-P residue. A well-known sign of endosome to lysosome maturation is gradual acidification (to pH ~5) in a mature lysosome; it is low endolysosomal pH that promotes dissociation of Man-6-P receptors from bound proteins; then, the receptors are transferred by transport vesicles back to the Golgi complex [95] or undergo hydrolysis in the lysosomal lumen.

There are no comprehensive data on the structural and functional organization of lysosomes and the mechanisms enabling the interaction of lysosomes with other cellular compartments. Furthermore, it is not entirely clear how the composition and functionality of lysosomes change throughout the cell life, as well as in different tissues and organs. In addition, according to some data, the lysosome pool is heterogeneous: these organelles apparently have different mechanisms for maintaining the internal acidic environment and receiving metabolic signals [107, 108]. These differences may be associated with the different positioning of lysosomes in cells, which is controlled by specialized protein complexes on the lysosome surface as well as by the activity of ion channels [109, 110].

**The role of lysosomes in human pathologies**

Many diseases are associated with a reduced activity of lysosomes and, therefore, with the accumulation of intracellular material (e.g., lipofuscin and ubiquitin); impaired activity of lysosomes is observed in age-related changes [111]. Several hereditary diseases, known as lysosomal storage diseases (LSD), are associated with defects in lysosomal enzymes. More than 50 different LSDs have been reported, which are caused by mutations in genes encoding lysosomal soluble hydrolases, membrane proteins, or auxiliary lysosomal proteins, something that leads to the blockage of a separate lysosomal catabolic pathway [112]. Accumulation of one main substrate is supposed to be caused by deficiency of a certain lysosomal enzyme. Now, this concept is the most popular; however, substantial evidence obtained from disease models and clinical studies seems to indicate that the LSD pathology is more complex than initially thought. The clinical manifestations of these diseases are heterogeneous: both systemic and neurological signs can occur at different ages and progress at different speeds. Breakdown of glycogen (glycogenosis), lipids (lipidosis) and proteoglycans (mucopolysaccharidosis) is impaired most often. Uncleaved macromolecules or degradation products accumulate in lysosomes and cause irreversible damage to cells over time. Organs increase in size, which leads to their dysfunction in severe cases. Typical examples of these diseases are Gaucher disease. associated with impaired glucocerebroside degradation; Tay-Sachs syndrome (impaired ganglioside degradation); and Pompe disease (impaired glycogen breakdown). The ability of MPR to recognize lysosomal enzymes modified by Man-6-P is considered as the basis for LSD enzyme replacement therapy [113]. Deficiency or mutations in lysosomal membrane proteins are also factors behind the development of many diseases. For example, an insufficient amount of the MCOLN1 protein causes type IV mucolipidosis [114]. CIC7 is associated with the development of osteopetrosis [115]. Mutations in the LAMP2A protein cause Danon’s disease, associated with the accumulation of autophagic vacuoles in muscle cells [116]. The Niemann- Pick C1 (NPC1) protein of the lysosomal membrane is involved in the export of cholesterol from the lysosome; mutations in this protein are considered to be the cause of the Niemann-Pick type C disease [117].

There is also ample evidence that lysosome dysfunction is one of the main mechanisms underlying the pathogenesis of neurodegenerative diseases, such as the Parkinson’s, Alzheimer’s and Huntington’s diseases [118, 119]. In addition, protein aggregates harmful to the cell can affect the efficiency of autophagy by inhibiting the recognition of cargo directed toward degradation by the autophagosome [120, 121].

## CONCLUSION

To date, it is obvious that, in addition to participating in degradation, ALS is directly involved in many important cellular processes, such as determining available nutrients, signal transmission, and regulation of cell metabolism. Despite the more than half-century history of studying this organelle, questions related to its structure and activity remain unclear. Systematic approaches, such as transcriptomics, proteomics, and metabolomics, combined with biochemical methods can help identify all components of the lysosome and expand our understanding of how ALS functions in general [111]. Unfortunately, little is known about how lysosomal functions change in different cells and tissues, at certain stages of cell development and in different organisms, as well as in changing physiological conditions. In addition, issues related to the existence of lysosomes with specialized functions, as well as the role of ALS in the pathogenesis of human diseases, such as impaired lipid metabolism, infection, and aging, remain open. Thoughtful and in-depth investigation of the functions of ALS will definitely take humanity to a qualitatively new level in the fight against many socially impactful diseases.

## Abbreviations

AD: Alzheimer’s disease
ADP: adenosine diphosphate
ALS: autophagic-lysosomal system
ATG: autophagy-related protein
ATP: adenosine triphosphate
CMA: chaperone-mediated autophagy
DUB: deubiquitinating enzyme
ER: endoplasmic reticulum
GTP: guanosine triphosphate
HD: Huntington’s disease
LAMP: lysosome-associated membrane protein
LIR: LC3-interacting region
LRO: lysosome-related organelle
mTORC1: mammalian target of rapamycin complex 1
PAS: phagophore assembly site
PD: Parkinson’s disease
PE: phosphatidylethanolamine
Ub: ubiquitin
UBD: ubiquitin-binding domain
UBL: ubiquitin-like
UPS: ubiquitin-proteasome system

## References

[1] Lilienbaum A. (2013). Int. J. Biochem. Mol. Biol..

[2] Hershko A., Ciechanover A., Varshavsky A. (2000). Nat. Med..

[3] Evnouchidou I., van Endert P. (2019). Hum. Immunol..

[4] Erales J., Coffino P. (2014). Biochim. Biophys. Acta..

[5] Murakami Y., Matsufuji S., Kameji T. (1992). Nature.

[6] Belogurov A., Kudriaeva A., Kuzina E., Smirnov I., Bobik T., Ponomarenko N., Kravtsova-Ivantsiv Y., Ciechanover A., Gabibov A. (2014). J. Biol. Chem..

[7] Kudriaeva A., Kuzina E.S., Zubenko O., Smirnov I. V., Belogurov A. (2019). FASEB J..

[8] Pierce N.W., Kleiger G., Shan S., Deshaies R.J. (2009). Nature.

[9] Kuma A., Mizushima N. (2010). Semin. Cell Dev. Biol..

[10] Kriegenburg F., Ungermann C., Reggiori F. (2018). Curr. Biol..

[11] Lamb C.A., Yoshimori T., Tooze S.A. (2013). Nat. Rev. Mol. Cell Biol..

[12] Grumati P., Dikic I. (2018). J. Biol. Chem..

[13] Mizushima N. (2018). Nat. Cell Biol..

[14] Wen X., Klionsky D.J. (2016). J. Mol. Biol..

[15] Bento C.F., Renna M., Ghislat G., Puri C., Ashkenazi A., Vicinanza M., Menzies F.M., Rubinsztein D.C. (2016). Annu. Rev. Biochem..

[16] Oku M., Sakai Y. (2018). BioEssays..

[17] Kaushik S., Cuervo A.M. (2012). Trends Cell Biol..

[18] Towers C.G., Thorburn A. (2016). EBioMedicine..

[19] Son S.M., Park S.J., Lee H., Siddiqi F., Lee J.E., Menzies F.M., Rubinsztein D.C. (2019). Cell Metab..

[20] Rogov V., Dötsch V., Johansen T., Kirkin V. (2014). Molecular Cell.

[21] Kirkin V., McEwan D.G., Novak I., Dikic I. (2009). Molecular Cell.

[22] Kamber R.A., Shoemaker C.J., Denic V. (2015). Molecular Cell.

[23] Lazarou M., Sliter D.A., Kane L.A., Sarraf S.A., Wang C., Burman J.L., Sideris D.P., Fogel A.I., Youle R.J. (2015). Nature.

[24] Stolz A., Ernst A., Dikic I. (2014). Nat. Cell Biol..

[25] Khaminets A., Behl C., Dikic I. (2016). Trends Cell Biol..

[26] Stolz A., Dikic I. (2014). Molecular Cell.

[27] Husnjak K., Dikic I. (2012). Annu. Rev. Biochem..

[28] Pankiv S., Clausen T.H., Lamark T., Brech A., Bruun J.-A., Outzen H., Øvervatn A., Bjørkøy G., Johansen T. (2007). J. Biol. Chem..

[29] Kirkin V., Lamark T., Sou Y.S., Bjørkøy G., Nunn J.L., Bruun J.A., Shvets E., McEwan D.G., Clausen T.H., Wild P. (2009). Molecular Cell.

[30] Korac J., Schaeffer V., Kovacevic I., Clement A.M., Jungblut B., Behl C., Terzic J., Dikic I. (2013). J. Cell Sci..

[31] Zhou J., Wang J., Cheng Y., Chi Y.J., Fan B., Yu J.Q., Chen Z. (2013). PLoS Genet..

[32] Lu K., Psakhye I., Jentsch S. (2014). Cell..

[33] Zheng Y.T., Shahnazari S., Brech A., Lamark T., Johansen T., Brumell J.H. (2009). J. Immunol..

[34] Thurston T.L.M., Ryzhakov G., Bloor S., von Muhlinen N., Randow F. (2009). Nat. Immunol..

[35] Wild P., Farhan H., McEwan D.G., Wagner S., Rogov V.V., Brady N.R., Richter B., Korac J., Waidmann O., Choudhary C. (2011). Science..

[36] Deosaran E., Larsen K.B., Hua R., Sargent G., Wang Y., Kim S., Lamark T., Jauregui M., Law K., Lippincott-Schwartz J. (2013). J. Cell Sci..

[37] Sarraf S.A., Raman M., Guarani-Pereira V., Sowa M.E., Huttlin E.L., Gygi S.P., Harper J.W. (2013). Nature.

[38] Wong Y.C., Holzbaur E.L.F. (2014). Proc. Natl. Acad. Sci. USA..

[39] Grasso D., Ropolo A., Lo Ré A., Boggio V., Molejón M.I., Iovanna J.L., Gonzalez C.D., Urrutia R., Vaccaro M.I. (2011). J. Biol. Chem..

[40] Marshall R.S., Li F., Gemperline D.C., Book A.J., Vierstra R.D. (2015). Molecular Cell.

[41] Pohl C., Jentsch S. (2009). Nat. Cell Biol..

[42] Guo H., Chitiprolu M., Gagnon D., Meng L., Perez-Iratxeta C., Lagace D., Gibbings D. (2014). Nat. Commun..

[43] Watson R.O., Manzanillo P.S., Cox J.S. (2012). Cell..

[44] Morimoto D., Walinda E., Fukada H., Sou Y.S., Kageyama S., Hoshino M., Fujii T., Tsuchiya H., Saeki Y., Arita K. (2015). Nat. Commun..

[45] Linares J.F., Duran A., Yajima T., Pasparakis M., Moscat J., Diaz-Meco M.T. (2013). Molecular Cell.

[46] Olzmann J.A., Li L., Chudaev M. V., Chen J., Perez F.A., Palmiter R.D., Chin L.-S. (2007). J. Cell Biol..

[47] Collins G.A., Goldberg A.L. (2017). Cell..

[48] Kalveram B., Schmidtke G., Groettrup M. (2008). J. Cell Sci..

[49] Cho S.J., Yun S.M., Jo C., Lee D., Choi K.J., Song J.C., Park S.I., Kim Y.J., Koh Y.H. (2015). Autophagy..

[50] Nakashima H., Nguyen T., Goins W.F., Chiocca E.A. (2015). J. Biol. Chem..

[51] Gamerdinger M., Kaya A.M., Wolfrum U., Clement A.M., Behl C. (2011). EMBO Rep..

[52] Pickford F., Masliah E., Britschgi M., Lucin K., Narasimhan R., Jaeger P.A., Small S., Spencer B., Rockenstein E., Levine B. (2008). J. Clin. Invest..

[53] Ravikumar B., Vacher C., Berger Z., Davies J.E., Luo S., Oroz L.G., Scaravilli F., Easton D.F., Duden R., O’Kane C.J. (2004). Nat. Genet..

[54] Winslow A.R., Chen C.W., Corrochano S., Acevedo-Arozena A., Gordon D.E., Peden A.A., Lichtenberg M., Menzies F.M., Ravikumar B., Imarisio S. (2010). J. Cell Biol..

[55] Lu K., Psakhye I., Jentsch S. (2015). Autophagy..

[56] Ohsumi Y., Ichimura Y., Kirisako T., Takao T., Satomi Y., Shimonishi Y., Ishihara N., Mizushima N., Tanida I., Kominami E. (2000). Nature.

[57] Shen Z., Li Y., Gasparski A.N., Abeliovich H., Greenberg M.L. (2017). J. Biol. Chem..

[58] Korolchuk V.I., Menzies F.M., Rubinsztein D.C. (2010). FEBS Lett..

[59] Jahreiss L., Menzies F.M., Rubinsztein D.C. (2008). Traffic..

[60] de Duve C. (2005). Nat. Cell Biol..

[61] Saftig P., Schröder B., Blanz J. (2010). Biochem. Soc. Trans..

[62] Olson O.C., Joyce J.A. (2015). Nat. Rev. Cancer..

[63] Luzio J.P., Parkinson M.D.J., Gray S.R., Bright N.A. (2009). Biochem. Soc. Trans..

[64] Li W., Yuan X., Nordgren G., Dalen H., Dubowchik G.M., Firestone R.A., Brunk U.T. (2000). FEBS Lett..

[65] Bursch W. (2001). Cell Death Differ..

[66] Guicciardi M.E., Leist M., Gores G.J. (2004). Oncogene..

[67] Leist M., Jäättelä M. (2001). Cell Death Differ..

[68] Leist M., Jäättelä M. (2001). Nat. Rev. Mol. Cell Biol..

[69] Chieregatti E., Meldolesi J. (2005). Nat. Rev. Mol. Cell Biol..

[70] Verhage M., Toonen R.F. (2007). Curr. Opin. Cell Biol..

[71] Rodríguez A., Webster P., Ortego J., Andrews N.W. (1997). J. Cell Biol..

[72] Andrews N.W. (2000). Trends Cell Biol..

[73] Jaiswal J.K., Andrews N.W., Simon S.M. (2002). J. Cell Biol..

[74] Luzio J.P., Hackmann Y., Dieckmann N.M.G., Griffiths G.M. (2014). Cold Spring Harb. Perspect. Biol..

[75] Stinchcombe J.C., Griffiths G.M. (2007). Annu. Rev. Cell Dev. Biol..

[76] Mostov K., Werb Z. (1997). Science..

[77] Logan M.R., Odemuyiwa S.O., Moqbel R. (2003). J. Allergy Clin. Immunol..

[78] Wesolowski J., Paumet F. (2011). Immunol. Res..

[79] Stinchcombe J., Bossi G., Griffiths G.M. (2004). Science..

[80] Ren Q., Ye S., Whiteheart S.W. (2008). Curr. Opin. Hematol..

[81] Rao S.K., Huynh C., Proux-Gillardeaux V., Galli T., Andrews N.W. (2004). J. Biol. Chem..

[82] Jahn R., Scheller R.H. (2006). Nat. Rev. Mol. Cell Biol..

[83] Bossi G., Griffiths G. (2005). Semin. Immunol..

[84] Cadwell K., Liu J.Y., Brown S.L., Miyoshi H., Loh J., Lennerz J.K., Kishi C., Kc W., Carrero J.A., Hunt S. (2008). Nature.

[85] DeSelm C.J., Miller B.C., Zou W., Beatty W.L., van Meel E., Takahata Y., Klumperman J., Tooze S.A., Teitelbaum S.L., Virgin H.W. (2011). Dev. Cell..

[86] Medina D.L., Fraldi A., Bouche V., Annunziata F., Mansueto G., Spampanato C., Puri C., Pignata A., Martina J.A., Sardiello M. (2011). Dev. Cell..

[87] Gerasimenko J.V., Gerasimenko O.V., Petersen O.H. (2011). Curr. Biol..

[88] Reddy A., Caler E. V., Andrews N.W. (2001). Cell..

[89] Roy D., Liston D.R., Idone V.J., Di A., Nelson D.J., Pujol C., Bliska J.B., Chakrabarti S., Andrews N.W. (2004). Science..

[90] Laplante M., Sabatini D.M. (2012). Cell..

[91] Sancak Y., Bar-Peled L., Zoncu R., Markhard A.L., Nada S., Sabatini D.M. (2010). Cell..

[92] Guertin D.A., Sabatini D.M. (2007). Cancer Cell..

[93] Settembre C., Zoncu R., Medina D.L., Vetrini F., Erdin S.S., Erdin S.S., Huynh T., Ferron M., Karsenty G., Vellard M.C. (2012). EMBO J..

[94] Schröder B.A., Wrocklage C., Hasilik A., Saftig P. (2010). Proteomics..

[95] Luzio J.P., Pryor P.R., Bright N.A. (2007). Nat. Rev. Mol. Cell Biol..

[96] Saftig P., Klumperman J. (2009). Nat. Rev. Mol. Cell Biol..

[97] Saftig P., Beertsen W., Eskelinen E.L. (2008). Autophagy..

[98] Alessandrini F., Pezzè L., Ciribilli Y. (2017). Semin. Oncol..

[99] Marshansky V., Rubinstein J.L., Grüber G. (2014). Biochim. Biophys. Acta - Bioenerg..

[100] Mindell J.A. (2012). Annu. Rev. Physiol..

[101] Chakraborty K., Leung K., Krishnan Y. (2017). Elife..

[102] Liu B., Du H., Rutkowski R., Gartner A., Wang X. (2012). Science..

[103] Efeyan A., Zoncu R., Sabatini D.M. (2012). Trends Mol. Med..

[104] Cang C., Zhou Y., Navarro B., Seo Y., Aranda K., Shi L., Battaglia-Hsu S., Nissim I., Clapham D.E., Ren D. (2013). Cell..

[105] Bagshaw R.D., Mahuran D.J., Callahan J.W. (2005). Mol. Cell. Proteomics..

[106] Coutinho M.F., Prata M.J., Alves S. (2012). Mol. Genet. Metab..

[107] Korolchuk V.I., Saiki S., Lichtenberg M., Siddiqi F.H., Roberts E.A., Imarisio S., Jahreiss L., Sarkar S., Futter M., Menzies F.M. (2011). Nat. Cell Biol..

[108] Johnson D.E., Ostrowski P., Jaumouillé V., Grinstein S. (2016). J. Cell Biol..

[109] Pu J., Schindler C., Jia R., Jarnik M., Backlund P., Bonifacino J.S. (2015). Dev. Cell..

[110] Li X., Rydzewski N., Hider A., Zhang X., Yang J., Wang W., Gao Q., Cheng X., Xu H. (2016). Nat. Cell Biol..

[111] Rubinsztein D.C., Mariño G., Kroemer G. (2011). Cell..

[112] Futerman A.H., van Meer G. (2004). Nat. Rev. Mol. Cell Biol..

[113] Neufeld E.F. (1980). Birth Defects Orig. Artic. Ser..

[114] Bach G., Bargal R., Avidan N., Ben-Asher E., Olender Z., Zeigler M., Frumkin A., Raas-Rothschild A., Glusman G., Lancet D. (2000). Nat. Genet..

[115] Sobacchi C., Schulz A., Coxon F.P., Villa A., Helfrich M.H. (2013). Nat. Rev. Endocrinol..

[116] Nascimbeni A.C., Fanin M., Angelini C., Sandri M. (2017). Cell Death Dis..

[117] Lloyd-Evans E., Morgan A.J., He X., Smith D.A., Elliot-Smith E., Sillence D.J., Churchill G.C., Schuchman E.H., Galione A., Platt F.M. (2008). Nat. Med..

[118] Menzies F.M., Fleming A., Rubinsztein D.C. (2015). Nat. Rev. Neurosci..

[119] Guo F., Liu X., Cai H., Le W. (2018). Brain Pathol..

[120] Orenstein S.J., Kuo S.H., Tasset I., Arias E., Koga H., Fernandez-Carasa I., Cortes E., Honig L.S., Dauer W., Consiglio A. (2013). Nat. Neurosci..

[121] Martinez-Vicente M., Talloczy Z., Wong E., Tang G., Koga H., Kaushik S., de Vries R., Arias E., Harris S., Sulzer D. (2010). Nat. Neurosci..

[122] Walkley S.U. (2009). J. Inherit. Metab. Dis..

